# Anaerobic Bacterial Immobilization and Removal of Toxic Sb(III) Coupled With Fe(II)/Sb(III) Oxidation and Denitrification

**DOI:** 10.3389/fmicb.2019.00360

**Published:** 2019-02-27

**Authors:** Jingxin Li, Yuxiao Zhang, Shiling Zheng, Fanghua Liu, Gejiao Wang

**Affiliations:** ^1^State Key Laboratory of Agricultural Microbiology, College of Life Science and Technology, Huazhong Agricultural University, Wuhan, China; ^2^Key Laboratory of Coastal Biology and Biological Resources Utilization, Yantai Institute of Coastal Zone Research, Chinese Academy of Sciences, Yantai, China

**Keywords:** *Sinorhizobium* sp., anaerobic Sb(III) oxidation, Fe(II) oxidation, Sb immobilization, denitrification

## Abstract

Antimony (Sb) pollution is a worldwide problem. In some anoxic sites, such as Sb mine drainage and groundwater sediment, the Sb concentration is extremely elevated. Therefore, effective Sb remediation strategies are urgently needed. In contrast to microbial aerobic antimonite [Sb(III)] oxidation, the mechanism of microbial anaerobic Sb(III) oxidation and the effects of nitrate and Fe(II) on the fate of Sb remain unknown. In this study, we discovered the mechanism of anaerobic Sb(III) oxidation coupled with Fe(II) oxidation and denitrification in the facultative anaerobic Sb(III) oxidizer *Sinorhizobium* sp. GW3. We observed the following: (1) under anoxic conditions with nitrate as the electron acceptor, strain GW3 was able to oxidize both Fe(II) and Sb(III) during cultivation; (2) in the presence of Fe(II), nitrate and Sb(III), the anaerobic Sb(III) oxidation rate was remarkably enhanced, and Fe(III)-containing minerals were produced during Fe(II) and Sb(III) oxidation; (3) qRT-PCR, gene knock-out and complementation analyses indicated that the arsenite oxidase gene product AioA plays an important role in anaerobic Sb(III) oxidation, in contrast to aerobic Sb(III) oxidation; and (4) energy-dispersive X-ray spectroscopy (EDS), X-ray photoelectron spectroscopy (XPS) and powder X-ray diffraction (XRD) analyses revealed that the microbially produced Fe(III) minerals were an effective chemical oxidant responsible for abiotic anaerobic Sb(III) oxidation, and the generated Sb(V) was adsorbed or coprecipitated on the Fe(III) minerals. This process included biotic and abiotic factors, which efficiently immobilize and remove soluble Sb(III) under anoxic conditions. The findings revealed a significantly novel development for understanding the biogeochemical Sb cycle. Microbial Sb(III) and Fe(II) oxidation coupled with denitrification has great potential for bioremediation in anoxic Sb-contaminated environments.

## Introduction

Antimony (Sb) is a metalloid that is widely used in a variety of industrial products (e.g., flame retardants, small arms ammunition, semiconductors and batteries) and medical treatments (e.g., for leishmaniasis) (Filella et al., 2002a; Vásquez et al., 2006; Wilson et al., 2010; He et al., 2012; Li et al., 2016). The increased mining, smelting and industry have significantly accelerated the emission of Sb into the environment, thereby leading to Sb accumulation in the human food chain (An and Kim, 2009; Hockmann et al., 2014). As suspected carcinogens, antimony species cause damage to many organ systems, such as the lung, heart, liver, and kidney (Sundar and Chakravarty, 2010). The United States Environmental Protection Agency and European Union have listed Sb compounds as priority pollutants (CEC, 1976; USEPA, 1979). Although there is much ongoing work to understand the speciation and relevant solution chemistry of Sb, many basic questions regarding Sb remain unclear, especially how it interacts with co-occurring elements and microbes. In nature, Sb mainly exists in two oxidation states, antimonite [Sb(III)] and antimonate [Sb(V)]. In aqueous environments at a neutral pH, Sb(III) is more prevalent in anoxic environments [as Sb(OH)_3_], and Sb(V) is dominate under oxic conditions [as Sb(OH)_6_] (Filella et al., 2002b; Hockmann et al., 2014). Sb(III) compounds are much more toxic than those containing Sb(V); thus Sb(III) oxidation, which transforms the toxic Sb(III) to the less toxic Sb(V), has a significant value for bioremediation of Sb-contaminated environments.

In addition to the chemical Sb(III) oxidants (e.g., H_2_O_2_, iodate, and Fe and Mn oxyhydroxides) (Belzile et al., 2001; Leuz and Johnsona, 2005; Quentel et al., 2006), microorganisms play an important role in Sb(III) oxidation, and this oxidation reaction serves as detoxification process for the microorganisms (Li et al., 2016). Bacteria can oxidize Sb(III) in both oxic and anoxic environments. In recent years, more than 60 aerobic Sb(III) oxidizers have been identified; among them, strains of *Pseudomonas*, *Comamonas*, *Agrobacterium*, and *Acinetobacter* genera comprise the majority (Li et al., 2013, 2016; Shi et al., 2013). Previously, we clarified the mechanisms of bacterial aerobic Sb(III) oxidation and found that Sb(III) oxidation is a co-metabolism process catalyzed by biotic (the antimonite oxidase AnoA and the arsenite oxidase AioAB) and abiotic (cellular H_2_O_2_) factors. The antimonite oxidase AnoA was discovered to catalyze Sb(III) oxidation in *Agrobacterium tumefaciens* GW4 with NADP^+^ as the co-factor (Li et al., 2015). The arsenite oxidase AioAB is composed of a large (AioA) and a small (AioB) subunit. Deletion of *aioA* partially decreased bacterial Sb(III) oxidation efficiency in *A. tumefaciens* 5A, while the function of *aioA* in Sb(III) oxidation in strain GW4 was not obvious (Wang et al., 2015; Li et al., 2017b). In addition, Sb(III) induces bacterial oxidative stress responses and produces substantial H_2_O_2_, and bacterially produced H_2_O_2_ acts as an oxidant to oxidize Sb(III) to Sb(V) (Li et al., 2017b). However, the knowledge of bacterial anaerobic Sb(III) oxidation is still limited. So far, only two anaerobic Sb(III) oxidizing strains of *Hydrogenophaga* and *Ensifer* have been isolated (Terry et al., 2015; Nguyen et al., 2017); these strains oxidize Sb(III) using nitrate as the electron acceptor, and the detailed mechanisms of these reactions remain unknown.

In mining areas, Sb frequently co-occurs with iron (Fe) and sulfur (S) (Filella et al., 2002a; Shi et al., 2013). Fe is the fourth most abundant element and the most redox-active metal in the Earth’s crust. In nature, Fe exists mainly as particulate ferric [Fe(III)], ferrous [Fe(II)] iron-bearing minerals or dissolved ions (Kappler and Straub, 2005). Fe(III) minerals have a high solubility at strongly alkaline or strongly acidic pH levels, while the solubility of Fe(III) minerals at circumneutral pH are extremely low. In contrast, some Fe(II) minerals are considerably more soluble at neutral pH (Kappler and Straub, 2005). In addition, Fe(II) can be oxidized to Fe(III) minerals by O_2_, but it persists in acidic pH even in oxic habitats (Cornell and Schwertmann, 2003). Fe(II) is more stable at neutral or alkaline pH in anoxic environments (Kappler and Straub, 2005). The Fe redox reactions are coupled with carbon, nitrogen, oxygen and sulfur cycles and are involved in important environmental processes such as methane oxidation, aromatic hydrocarbon degradation, heavy metal passivation and immobilization (Vodyanitskii, 2010; Niedźwiecka et al., 2017; Yan et al., 2018; Yuan et al., 2018). Many studies have documented that Fe(II) oxidation is closely associated with the fate of heavy metals in anoxic environments (Vodyanitskii, 2010). It is known that circumneutral pH environments are prevailing on Earth, and the pH of many heavy metal-polluted sites (e.g., mining and shooting areas) are also near neutral (Okkenhaug et al., 2013; Shi et al., 2013). However, in anoxic neutral habitats, only Mn(IV) and nitrite can oxidize Fe(II) chemically (Kappler and Straub, 2005; Melton et al., 2014). It was reported that Fe(II) oxidation is mainly a biotical process mediated by nitrate dependent anaerobic ferrous oxidizing bacteria (NAFOB) under anoxic conditions at neutral pH (Kappler and Straub, 2005; Melton et al., 2014).

NAFOB oxidation of Fe(II) to Fe(III) is coupled with nitrate reduction, which produces Fe(III) minerals and ammonium, nitrogen or denitrification intermediates (Coby et al., 2011; Nordhoff et al., 2017; Schaedler et al., 2018). Thus, NAFOB play an important role in the process of biomineralization and heavy metal passivation, and thereby affect the migration of heavy metals such as chromium (Cr), arsenic (As), and Sb (Senn and Hemond, 2002; Richmond et al., 2004; Vodyanitskii, 2010). Currently, the Fe-N-As coupling mechanism mediated by NAFOB has been found in anoxic environments (Hohmann et al., 2009). Chen et al. (2008) demonstrated that nitrate-dependent Fe(II) oxidation in paddy soil reduced the bioavailability of As through coprecipitation and/or adsorption to Fe(III) minerals. Additionally, the nitrate-dependent, Fe(II)-oxidizing bacteria *Acidovorax* sp. BoFeN1 isolated from freshwater sediment effectively immobilize more than 96% of soluble As(III) or As(V) in the presence of Fe(II) (Hohmann et al., 2009). However, compared with anaerobic As(III) oxidation, the role of NAFOB in anaerobic Sb(III) oxidation and immobilization remains poorly understood.

The aim of this study was to clarify the coupling mechanism of Fe-N-Sb under anoxic conditions in the facultative-anaerobic, Sb(III)- and Fe(II)-oxidizing strain *Sinorhizobium* sp. GW3 (Fan et al., 2008). We provide comprehensive evidence showing that Fe(II) and nitrate have a strong influence on the transformation of Sb species and mobility in anoxic environments. This study represents a novel contribution to understanding the Sb biogeochemical cycle and provides a potential bioremediation strategy for anoxic environmental Sb contamination.

## Materials and Methods

### Strains and Genomic Analysis

The facultative anaerobic bacterium *Sinorhizobium* sp. GW3 (Genome GenBank No. AUSY00000000.1) was mainly used in this study. This strain was isolated by our research group from arsenic contaminated groundwater sediments in Shanyin City, Shanxi Province, China. It could oxidize As(III) aerobically (Fan et al., 2008). The anaerobic As(III) oxidation of strain GW3 was performed using KMnO_4_ method as described by Cai et al. (2009). For *aioA* gene knock-out and complementation study, strain *A. tumefaciens* GW4 was used (Fan et al., 2008; Wang et al., 2015). Genomic analyses of putative genes involved in denitrification, As(III)/Sb(III) oxidation/resistance, anaerobic selenite-, sulfate-, iron- and chromate- reduction and transportation were conducted through BlastN and BlastP in the genome of *Sinorhizobium* sp. GW3 on the NCBI website^1^.

### Aerobic Sb(III) Oxidation Assays

For aerobic Sb(III) oxidation experiments, bacterial strains were grown in chemically defined medium (CDM) (Weeger et al., 1999) containing 0 or 20 μM K_2_Sb_2_(C_4_H_2_O_6_)_2_ [Sb(III)] with shaking at 28°C. Overnight cultures of *Sinorhizobium* sp. GW3 strain were inoculated into 5 mL of CDM in the presence of 20 μM Sb(III) and incubated at 28°C with 120 rpm shaking. When the OD_600_ reached 0.5–0.6, the cultures were each inoculated into 100 mL of CDM with 20 μM Sb(III) and different amounts (0, 1, and 5 mM) of KNO_3_^−^. At designated times, the culture samples were centrifuged (13,400 × *g*, 5 min) and subsequently filtered (0.22 μm filter) to measure Sb(III) oxidation and denitrification. The Sb(III)/Sb(V) content was measured by HPLC-HG-AFS (Beijing Titan Instruments Co., Ltd., China) (Li et al., 2013), and the concentration of NO_3_^−^/NO_2_^−^ was measured by HPLC (HPLC 2690 series, Waters, MA, United States) according to the method described by Croitoru (2012).

### Anaerobic Sb(III) Oxidation Using Nitrate as the Electron Acceptor

For denitrification and anaerobic Sb(III) oxidation experiments, bacterial strains were cultured in CDM with 0 or 100 μM Sb(III) under a gas phase of N_2_/CO_2_ (80/20). The cultures were incubated at 28°C without shaking, and all operations were carried out in an anaerobic chamber (Vinyl Glove Box, Coy Laboratory Products) (Wang et al., 2018). First, strain GW3 was cultured anaerobically in CDM with 1 mM nitrate to investigate whether nitrate could be used as the electron acceptor for anaerobic growth. Samples were taken regularly to measure the NO_3_^−^ and NO_2_^−^ content. Then, bacterial cells were harvested by centrifugation (6,000 × *g*, 5 min) and washed twice with anoxic physiological saline after 10 days of cultivation. The resuspension was used as an inoculum for anaerobic Sb(III) oxidation. For nitrate addition, we used 1 mM KNO_3_, which is similar to the amount of nitrate used in anaerobic As(III) oxidation (Zhang et al., 2017). To further investigate the effect of nitrate on anaerobic Sb(III) oxidation, different concentrations (0, 1, and 5 mM) of nitrate were added to the culture. Additionally, to observe the nitrate-induced oxidation of anaerobic Sb(III), 100 μM Sb(III) was used for the Sb(III) oxidation analysis instead of 20 μM Sb(III). Thus, the rate of anaerobic Sb(III) oxidation was determined in CDM amended with 100 μM Sb(III) and 0, 1, or 5 mM nitrate as the electron acceptor. The serum bottles (100 mL) containing 50 mL of CDM were flushed with a N_2_ gas stream and sealed with butyl rubber stoppers and aluminum covers. After autoclaving, the stock cultures were each inoculated into the sealed serum bottles via injection to a final OD_600_ of 0.2, and the cultures were incubated at 28°C without shaking. At designated times, 100 μL of culture was withdrawn anoxically with a syringe for measuring the Sb(III)/Sb(V) and NO_3_^−^/NO_2_^−^ content, as described above.

### Anaerobic Fe(II) Oxidation Using Nitrate as the Electron Acceptor

For anaerobic Fe(II) oxidation assays, stock cultures of strain GW3 were each inoculated into 50 mL of CDM containing 1 or 5 mM nitrate and 1 mM Fe(II). The FeCl_2_ stock liquid was prepared in an anaerobic chamber and subsequently injected into the culture. Strain GW3 was inoculated as described above. The experiment was performed in triplicate with no GW3 inoculum as a control, and all cultures were incubated at 28°C in the dark. The samples were taken periodically to determine the degree of Fe(II) oxidation and nitrate reduction. The dissolved Fe(II) content was quantified by the ferrozine colorimetric assay at 562 nm using an EnVision^®^ Multimode Plate Reader (PerkinElmer) (Stookey, 1970).

### Gene Expression Analyses

To investigate the expression of genes involved in Sb(III) oxidation and resistance under oxic conditions, overnight cultures of strain GW3 were inoculated into 100 mL of CDM with 0 or 100 μM Sb(III) at 28°C with 120 rpm shaking. After 24 h of incubation, bacterial cells were harvested for total RNA extraction. For anoxic gene expression analysis, bacterial cells were each inoculated into oxygen-free CDM with 0 or 100 μM Sb(III) and incubated at 28°C without shaking. After incubation for 4 days, bacterial cells were harvested and treated with Trizol reagent (Invitrogen).

Total RNA was extracted according to the manufacturer’s instructions (Invitrogen). Genomic DNA was removed by treatment with RNase-free DNase I (Takara) at 37°C, and the concentration of RNA was monitored using a spectrophotometer (NanoDrop 2000, Thermo). Reverse transcription was performed with 300 ng of total RNA for each sample using the RevertAid First Strand cDNA Synthesis Kit (Thermo) (Wang et al., 2012). Subsequently, the obtained cDNA was diluted 10-fold and used as a template for qRT-PCR, which was carried out in an ABI VIIA7 system in 0.1 mL Fast Optical 96-well Reaction Plates (ABI). The primers used for qRT-PCR are listed in Supplementary Table S1. Each reaction was replicated three times to estimate error. The 16S rRNA gene of strain GW3 was used as an internal control. First, standard dilution curves were generated for each pair of primers. Then, qRT-PCR was conducted to obtain *C*t values for each sample. The slope (*S*) of each standard curve was used to calculate the primer amplification efficiency (*E*) with [log (1+*E*) = 1/*S*]. The *C*t values of target cDNAs were each normalized with the cDNA *C*t value of 16S rRNA to generate the Δ*C*t value of each sample in the control and Sb(III)-treatment groups. Subsequently, the Δ*C*t values of cDNAs in the control groups were each subtracted from those in the Sb(III) treatment groups. The relative gene expression ratios were analyzed using the 2^−ΔΔCT^ method (Livak and Schmittgen, 2001). Significance analysis was performed by one-way ANOVA.

To investigate the role of *aioA* in anaerobic Sb(III) oxidation, *A. tumefaciens* GW4 and the corresponding *aioA*-deletion and *aioA*-complementation strains were used to determine the anaerobic Sb(III) oxidation efficiency. *A. tumefaciens* strains were each inoculated into 50 mL of CDM containing 1 mM nitrate and 100 μM Sb(III) under anoxic conditions and cultured as described above. At designated times, samples were taken for measuring the Sb(III)/Sb(V) content.

### Effects of Fe(II) on Anaerobic Sb(III) Oxidation and Immobilization in the Presence of Nitrate

A stock culture of strain GW3 was inoculated into 50 mL of CDM with 1 mM Fe(II), 100 μM Sb(III) and 1 mM nitrate under anoxic conditions. CDM containing 100 μM Sb(III) and 1 mM nitrate was used as a control. All cultures were incubated for up to 10 days at 28°C in the dark without shaking. Changes in Sb(III), Fe(II) and nitrate concentrations were determined as described above. Precipitates from anaerobic Fe(II)- and Sb(III)-oxidizing cultures were collected at the end of the experiment by centrifugation (13,400 × *g*, 5 min) and freeze-dried overnight. The elemental composition of the samples was investigated by energy-dispersive X-ray spectroscopy (EDS) using an Oxford Instruments model Inca X-sight EDS detector, which is coupled with the SEM (FEI Inspect F50). Powder X-ray diffraction (XRD) analysis was conducted using a Bruker D8 Advance X-ray diffractometer with Cu Kα as the X-ray source (40 mA, 20 kV). The elemental chemical status and compositions were analyzed with X-ray photoelectron spectroscopy (XPS) using Al Kα (1486.6 eV) excitation.

### Anaerobic Sb(III) Oxidation by Microbially Produced Fe(III) Minerals

Bacterial cells were cultured as described above. Stock cultures of strain GW3 were inoculated into 50 mL of CDM containing 1 mM nitrate and 1 mM Fe(II) under anoxic conditions. After 10 days of static cultivation at 28°C in the dark, the orange precipitates produced by strain GW3 were harvested through centrifugation (13,400 × *g*, 5 min); these precipitates included both Fe(III) minerals and bacterial cells. Subsequently, the mixture was resuspended in PBS (pH 7.0) and injected into oxygen-free CDM containing 100 μM Sb(III). Meanwhile, the precipitates were taken immediately, washed three times with PBS, and used for plate counting to detect the bacterial numbers. The bacteria cultured without Fe(II) were used as a control. During cultivation, 100 μL of culture was withdrawn anoxically with a syringe for measuring the Sb(III) content at designated times. To test whether nitrite or Fe(III) could oxidize Sb(III) abiotically, 50 mL of CDM was added to the serum bottles under an atmosphere of N_2_. After sterilization and sealing, 1 mM NaNO_2_ or 1 mM FeCl_3_ were amended with 100 μM Sb(III) and incubated for up to 10 days at 28°C under anoxic conditions. Samples were regularly taken to monitor the Sb(III)/Sb(V) and NO_2_^−^ content.

## Results

### Genomic Analysis of *Sinorhizobium* sp. GW3

Strain GW3 is a facultative anaerobic bacterium which can oxidize As(III) under both oxic and anoxic conditions (Fan et al., 2008; Supplementary Figure S1). The genome of strain GW3 contains putative genes responsible for aerobic As(III) and Sb(III) oxidation [the As(III) oxidase genes *aioAB*, Sb(III) oxidase gene *anoA* and catalase gene *katA*] (Supplementary Table S2). However, it has no *arxA* gene, which encodes anaerobic As(III) oxidase (Zargar et al., 2012). The genes involved in As(III) and Sb(III) resistance were also found, such as As(III)/Sb(III) efflux gene *acr3* and As(V) reductase gene *arsC* (Rosen, 1995; Supplementary Table S2). These As(III)/Sb(III) oxidation and resistance genes are all single copy. It has been reported that the multicopper oxidase may play a role in bacterial anaerobic Fe(II) oxidation (He et al., 2016). Expectably, we found three multicopper oxidase genes in strain GW3 (Supplementary Table S2). In addition, there are also genes involved in denitrification and sulfate reduction. Interestingly, the strain GW3 genome also harbors genes which might contribute to anaerobic selenite reduction, iron reduction and chromate reduction (Supplementary Table S2), suggesting that strain GW3 may have variable anaerobic functions.

### The Addition of Nitrate Has no Effect on Bacterial Aerobic Sb(III) Oxidation

**FIGURE 1:**
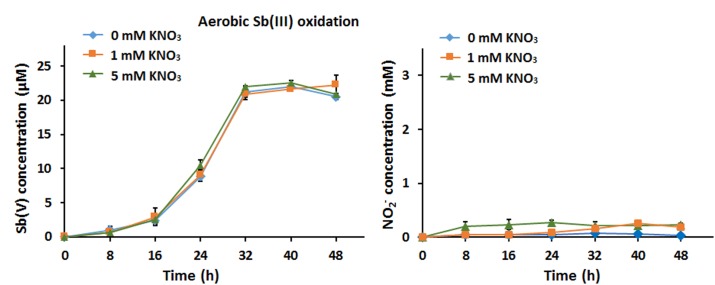
Nitrate had no effect on bacterial aerobic Sb(III) oxidation. **(A)** Sb(III) oxidation profiles of *Sinorhizobium* sp. GW3 with the addition of 20 μM Sb(III) and different concentrations of nitrate under oxic conditions. Sb(V) concentrations in the culture fluids were measured using HPLC-HG-AFS. **(B)** Nitrite production by strain GW3 during the process of aerobic Sb(III) oxidation. Nitrite concentrations in the culture fluids were measured using HPLC. Error bars represent standard deviations of the means from three independent experiments.

We first assessed the effect of nitrate addition on bacterial Sb(III) oxidation under oxic conditions. Strain GW3 was inoculated into CDM containing 20 μM Sb(III) and different concentrations (0, 1, and 5 mM) of nitrate, and the culture was aerated by shaking at 28°C. As shown in Figure 1A, strain GW3 could oxidize Sb(III) aerobically; however, there was no significant difference in Sb(III) oxidation rates upon the addition of different concentrations of nitrate (Figure 1A). Nitrate reduction experiments showed that nitrate was only slightly reduced to nitrite regardless of the concentration of nitrate added (Figure 1B). These results suggested that nitrate has no effect on bacterial aerobic Sb(III) oxidation and strain GW3 tends to use O_2_ as the electron acceptor rather than nitrate under oxic conditions.

### Nitrate Is the Electron Acceptor in Anaerobic Sb(III) Oxidation

**FIGURE 2:**
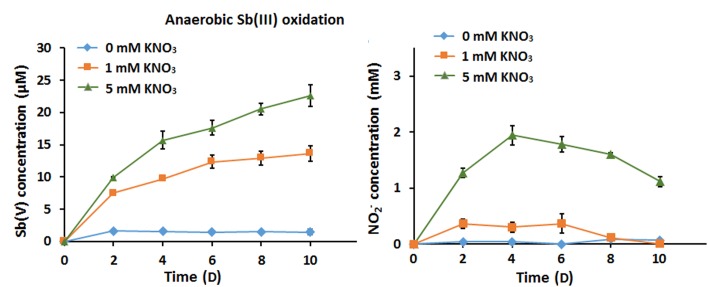
Effects of nitrate addition on bacterial anaerobic Sb(III) oxidation. **(A)** Sb(III) oxidation profiles of *Sinorhizobium* sp. GW3 with 100 μM Sb(III) and different concentrations of nitrate under anoxic conditions. **(B)** Nitrite formation by strain GW3 during the anaerobic Sb(III) oxidation process. Error bars represent standard deviations of the means from three independent experiments.

Under anoxic conditions in CDM, strain GW3 could reduce NO_3_^−^ to NO_2_^−^, and the contents of NO_2_^−^ decreased (Supplementary Figure S2A); this suggests that the NO_2_^−^ produced flowed into subsequent denitrification processes to generate N_2_O or N_2_, consistent with the presence of *nir*, *nor*, and *nos* genes in the strain GW3 genome (Supplementary Table S2). Upon addition of Sb(III), the denitrification efficiency increased compared to the efficiency in conditions without Sb(III); this was apparent because the amount of NO_2_^−^ produced with 1 mM nitrate and 100 μM Sb(III) was less than that produced with 1 mM nitrate only (Supplementary Figure S2B). This suggests that the denitrification and Sb(III) oxidation processes enhance electron transfer. Thus, the Sb(III) oxidation rate was significantly increased by the greater amounts of nitrate (Figure 2A). We did not observe any abiotic Sb(III) oxidation by nitrite (Supplementary Figure S3A), and the content of nitrite was constant during 10 days of cultivation (data not shown). Additionally, we tested whether Sb(III) could be oxidized abiotically by FeCl_3_. The results showed that the Sb(III) content was not decreased and Sb(V) was not observed in the presence of FeCl_3_ (Supplementary Figure S3B). The results indicated that the intermediate products of denitrification in strain GW3 and FeCl_3_ could not oxidize Sb(III) abiotically.

### Transcription Levels of Genes Involved in Sb(III) Oxidation Under Oxic and Anoxic Conditions

To investigate the potential mechanism of Sb(III) oxidation in strain GW3, the transcript levels of *aioA*, *anoA*, and *katA* were measured under both oxic and anoxic conditions using qRT-PCR. Additionally, we assessed the transcript levels of the As(III)/Sb(III)-resistance genes *acr3* and *arsC*. The original qRT-PCR data and primer efficiency are shown in Supplementary Table S3 and Supplementary Figure S3, respectively. Since the primer amplification efficiencies for 16S rRNA and other genes were similar (∼5%) (Supplementary Figure S4), primer amplification efficiencies were not included in the calculation of the relative gene expression ratios.

**FIGURE 3:**
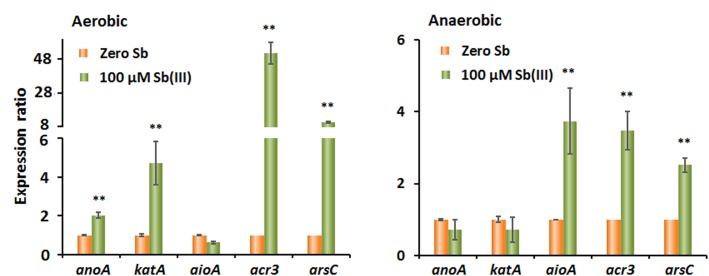
Quantitative reverse transcription-PCR analysis of the genes associated with Sb(III) oxidation and resistance in strain GW3 under both aerobic **(A)** and anaerobic **(B)** conditions. Total RNA was isolated from strain GW3 cultured with or without 100 μM Sb(III). The 16S rRNA gene was used as a reference. Data are shown as the mean of three replicates, with the error bars representing ± SD. ^∗∗^ Represents *p* < 0.01; ^∗^ represents *p* < 0.05.

The results showed that the transcript levels of *anoA*, *katA*, *acr3*, and *arsC* all significantly increased upon the addition of Sb(III), while the transcript level of *aioA* was not increased by Sb(III) under oxic conditions (Figure 3A). These findings hint that the gene products of *anoA* and the cellular oxidative stress response may substantially contribute to aerobic Sb(III) oxidation in strain GW3, which is consistent with our previous observations in *A. tumefaciens* GW4 (Li et al., 2015, 2017a,b). In contrast, under anoxic conditions, the addition of Sb(III) had no significant effect on the transcript levels of *anoA* and *katA*, while *aioA*, *acr3*, and *arsC* levels were promoted by Sb(III) (Figure 3B). The results suggested that AioA may play a role in bacterial anaerobic Sb(III) oxidation, and Acr3 and ArsC may be involved in Sb(III) resistance under both oxic and anoxic conditions.

To further analyze the role of *aioA* in anaerobic Sb(III) oxidation, strain *A. tumefaciens* GW4 and the corresponding *aioA*-deletion and *aioA*-complementation strains were used in the anaerobic Sb(III) oxidation experiments. Strain GW4 is closely related to strain GW3 based on the phylogenetic analysis of the 16S rRNA genes (Fan et al., 2008); furthermore, the amino acid sequence similarity of AioA in these two strains is 77%. We found that the oxidation efficiency of GW4-Δ*aioA* was 73% lower than that of wild-type strain GW4, and the complementary strain rescued the oxidation efficiency phenotype (Supplementary Figure S5). This suggests that AioA contributes to bacterial anaerobic Sb(III) oxidation in strain GW4 and may also contribute in the closely related strain GW3.

### The Addition of Nitrate Enhanced Anaerobic Fe(II) Oxidation

**FIGURE 4:**
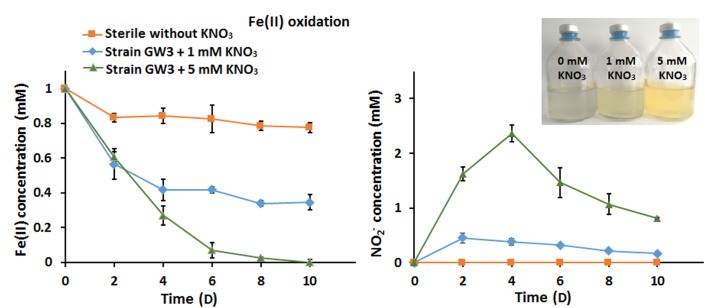
Effects of nitrate on bacterial anaerobic Fe(II) oxidation. **(A)** Fe(II) oxidation profiles of *Sinorhizobium* sp. GW3 with 1 mM Fe(II) and different concentrations of nitrate under anoxic conditions. No inoculum was added to the sterile control. **(B)** Nitrite formation by strain GW3 during the anaerobic Fe(II) oxidation process. Error bars represent standard deviations of the means from three independent experiments.

The ability of strain GW3 to oxidize Fe(II) under anaerobic nitrate-reducing conditions was also tested. The Fe(II) stock culture was prepared anaerobically in an anaerobic chamber and then injected into the culture. Compared with the sterile control, the bacterial Fe(II) oxidation rate increased as the concentration of added nitrate increased (Figure 4A). At the highest concentration of nitrate tested (5 mM nitrate), all of the Fe(II) was consumed within 10 days (Figure 4A), indicating that 5 mM nitrate could accept all electrons from 100 μM Fe(II) oxidation. Correspondingly, the NO_2_^−^ content increased first and subsequently decreased (Figure 4B), consistent with the trend of nitrate reduction during anaerobic Sb(III) oxidation (Figure 2B). The addition of nitrate significantly stimulates bacterial Fe(II) oxidation, indicating that strain GW3 is also a nitrate-dependent, anaerobic ferrous-oxidizing bacterium. Additionally, orange precipitates were observed during cultivation (Figure 4B), indicating that Fe-containing minerals were formed during the Fe(II) oxidation process.

### Fe(II) Stimulated Anaerobic Sb(III) Oxidation and Immobilization in the Presence of Nitrate

**FIGURE 5:**
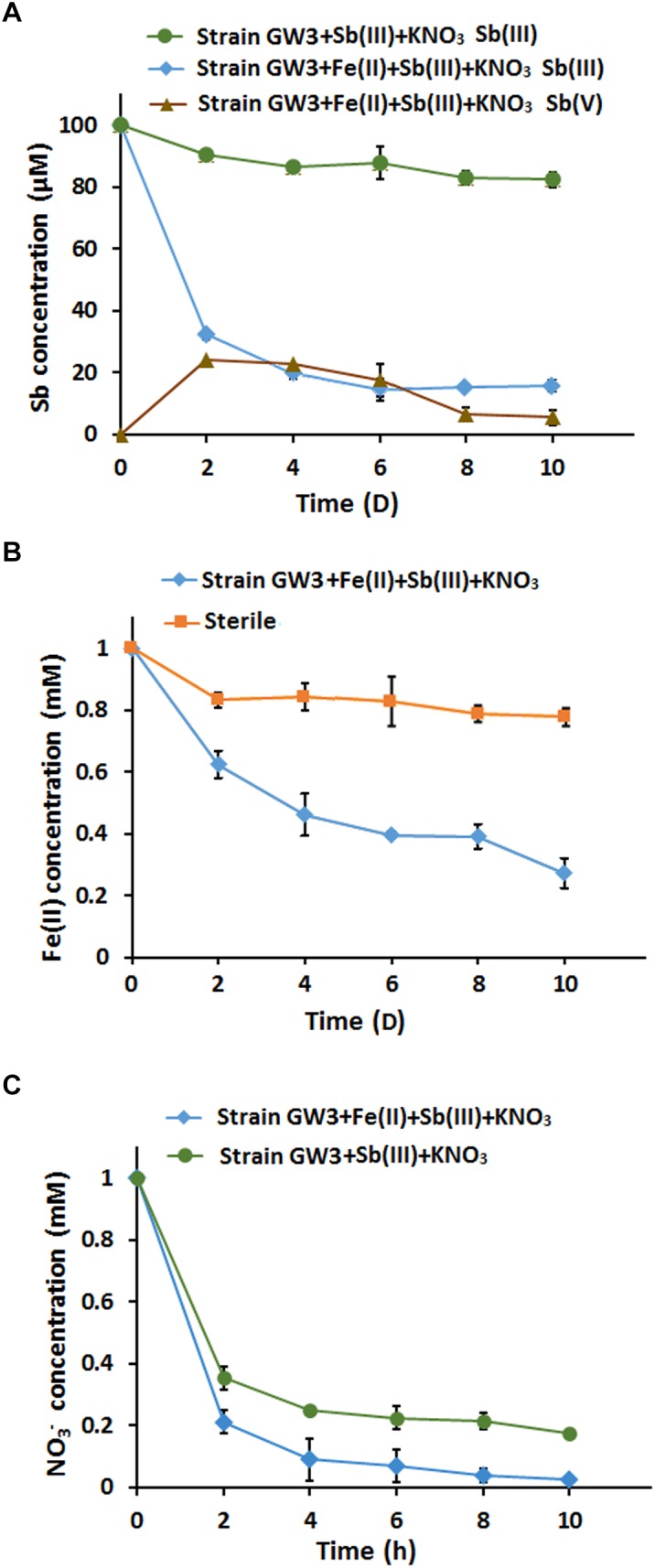
The addition of FeCl_2_ enhanced bacterial anaerobic Sb(III) oxidation and immobilization in the present of nitrate. **(A)** Sb(III) oxidation curves. **(B)** Fe(II) oxidation curves. A sterile culture without inoculation of strain GW3 was used as a control. **(C)** Nitrate consumption curves. Error bars represent standard deviations of the means from three independent experiments.

We assessed the effects of Fe(II) on bacterial anaerobic Sb(III) oxidation in CDM containing 100 μM Sb(III) and 1 mM nitrate with or without 1 mM Fe(II) under anoxic conditions. Sb(III) oxidation experiments showed that up to approximately 15.9 μM Sb(III) was reduced by the end of 10 days of cultivation in the presence of Sb(III) and nitrate, and 82.4 μM Sb(III) was reduced when Fe(II) was added (Figure 5A); Fe(II) significantly increased bacterial Sb(III) oxidation efficiency by 66.5%. Interestingly, the Sb(V) content increased first and subsequently decreased, and orange precipitates were observed in the culture with Fe(II). The increase in Sb(V) concentration did not compensate for the decrease in Sb(III) concentration, suggesting that Sb may adsorb or coprecipitate on the Fe-containing minerals. The Fe(II) oxidation experiment showed that up to approximately 0.75 mM Fe(II) was consumed by the end of 10 days of cultivation, and this value was significantly lower than the sterile control (Figure 5B). As the electron acceptor, nitrate was reduced to nitrite and other denitrification intermediates. Fe(II) addition accelerated the consumption of nitrate, since Fe(II) oxidation also needs nitrate as an electron acceptor (Figure 5C).

To investigate the chemical components of the precipitate, XRD analysis was conducted. However, we did not identify any characteristic peaks, indicating that the secondary mineral formed by strain GW3 was poorly crystalline (Supplementary Figure S6A). Thus, XPS analysis was further employed to determine the valence state of elements. The results showed that the valence state of iron mineral produced by strain GW3 in the presence of Fe(II) and nitrate was positive trivalent (Fe2p peak at 711.25 eV; Supplementary Figure S6B), indicating that strain GW3 could produce Fe(III)-containing minerals during anaerobic Fe(II) oxidation. In the presence of Fe(II), Sb(III) and nitrate together, the XPS spectra showed Fe2p and Sb3p peaks at 711.05 (Supplementary Figure S6C) and 531.71 eV (Supplementary Figure S6D), respectively, which were attributed to Fe(III) and Sb(V), respectively (Wu et al., 2018). These observations indicated that the removed Sb(III) was its oxidized product Sb(V) on the Fe(III)-containing minerals.

### Microbially Produced Fe(III)-Containing Minerals Efficiently Oxidized and Immobilized Sb(III)

To identify the role of microbially produced Fe(III)-containing minerals in anoxic Sb(III) oxidation, the orange precipitates were harvested after 10 days of cultivation and then added to CDM in the presence of 100 μM Sb(III) under anoxic conditions. Since the precipitates need to be maintained in their original state with their original chemical properties, no extra steps were taken to remove cells. To determine whether there were living bacteria in the Fe(III)-containing mineral precipitates, a plate counting assay was employed to compare the number of bacteria in the precipitates and the control cells after 10 days of cultivation. The results showed that there was no difference in the cell counts between the precipitates and the cultures with only cells, and no Sb(III) oxidation was observed in the supernatant of the culture (data not shown).

**FIGURE 6:**
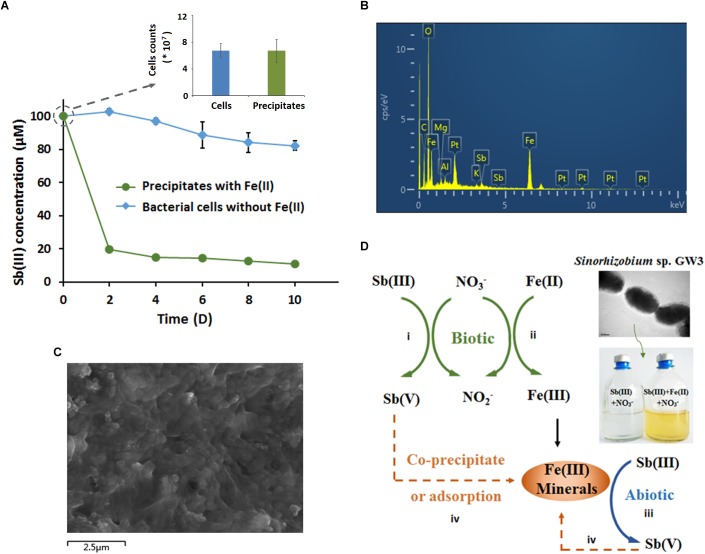
Microbially produced Fe(III)-containing minerals enhanced Sb(III) oxidation and immobilization **(A–C)**, and a proposed model of the Fe-N-Sb coupling mechanism mediated by strain GW3 **(D)**. **(A)** Sb(III) oxidation curves of the microbially produced Fe(III)-containing minerals. Strain GW3 was anaerobically inoculated into CDM containing 1 mM KNO_3_ with or without 1 mM Fe(II). After 10 days of cultivation, the precipitate was harvested and added to fresh CDM in the presence of 100 μM Sb(III). Meanwhile, the precipitate was washed three times with PBS and then used for plate counting to detect the number of bacteria. Error bars represent standard deviations of the means from three independent experiments. **(B,C)** EDS analysis and SEM image of the Fe(III)-containing minerals after 10 days of cultivation under the conditions described in **(A)**. The EDS peaks for Al, Mg, and Pt may come from aluminum alloy on the sample table and the spraying Pt treatment, which is used to increase electrical conductivity.

To compare the contribution of biotic and abiotic Sb(III) oxidation, bacterial cells cultured without Fe(II) were also collected as a control. Compared with the control cells without precipitates, the amount of Sb(III) significantly decreased in the culture with microbially produced Fe(III)-containing precipitates (Figure 6A). However, the increased Sb(III) oxidation efficiency is due to the function of both Fe(III)-containing minerals (abiotic) and bacterial cells (biotic). As shown in Figure 6A, after 10 days of cultivation, the Sb(III) oxidation efficiency of bacterial cells was 18% (the biotic effort), while the Sb(III) oxidation efficiency of the precipitates which included Fe(III)-containing minerals and bacterial cells was 89%. Thus, the contribution of abiotic Sb(III) oxidation in this system was approximately 71%.

The precipitates were harvested for SEM and EDS analyses. Consistent with the previous results, the precipitates contained Fe and Sb (Figure 6B), indicating that the microbially produced Fe(III)-containing minerals greatly contributed to the increased anaerobic Sb(III) oxidation and the immobilization of Sb(V). As shown in Figure 6C, the surface of the precipitate was porous, which provided a sorption phase for Sb(V) immobilization. Additionally, Sb(III) could not be oxidized abiotically by FeCl_3_, indicating that the artificial Fe(III) solution without secondary mineral formation from microbes has no functional roles in Sb(III) oxidation.

## Discussion

Sb(III) oxidation by oxygen is naturally very slow (Leuz and Johnsona, 2005), and Sb(III) can readily precipitate with sulfide or be strongly adsorbed by Fe(III) hydroxides at neutral pH under anoxic environments (Lai et al., 2016). Thus, microorganisms play an important role in Sb(III) oxidation (Li et al., 2016). Compared to the increasing knowledge of bacterial aerobic Sb(III) oxidation, there are only two known bacteria capable of anaerobic Sb(III) oxidation to date (Terry et al., 2015; Nguyen et al., 2017). We observed that during anaerobic cultivation, the bacterial denitrification efficiency was enhanced by Sb(III) since Sb(III) provides more electrons besides the electron donors in the CDM (such as the sodium lactate); meanwhile, the anaerobic Sb(III) oxidation efficiency also increased significantly as the concentration of nitrate increased. Thus, in the presence of nitrate and Sb(III) oxidizing bacteria, the rate of Sb(III) oxidation is enhanced greatly under anoxic environments and thereby accelerates the biogeochemical cycle of Sb.

We previously elucidated the co-metabolism mechanism of bacterial aerobic Sb(III) oxidation (Wang et al., 2015; Li et al., 2016, 2017a,b). However, there is no evidence describing the anaerobic Sb(III) oxidation mechanism. In this study, based on the qRT-PCR and gene knock-out and complementation analyses, we show that the bacterial Sb(III) oxidation mechanisms differ in oxic and anoxic conditions. The AnoA catalysis and active oxygen generation tend to occur under oxic conditions. Under anoxic conditions, AioA plays a role in anaerobic bacterial Sb(III) oxidation. Additionally, Acr3 and ArsC are involved in Sb(III) resistance under both oxic and anoxic conditions.

Interestingly, *Sinorhizobium* sp. GW3 is also a nitrate-dependent Fe(II)-oxidizing strain. The addition of nitrate significantly increased bacterial anaerobic Fe(II) oxidation and produced Fe(III)-containing precipitate, which would provide a sorption phase for metal(loid)s. These findings are consistent with previous studies of *Acidovorax* sp. ST3 isolated from As-contaminated paddy soils (Zhang et al., 2017). As expected, we confirmed that Sb(III) could be efficiently oxidized and immobilized by the microbially produced Fe(III)-containing precipitate. Thus, we propose that the coupling mechanism of Fe-N-Sb driven by strain GW3 under anoxic conditions is a combination of biotic and abiotic redox reactions, and abiotic Sb(III) oxidation is more efficient than biotic Sb(III) oxidation (Figure 6D). This is the first demonstration of the coupling mechanism of Fe-N-Sb driven by a nitrate-dependent Fe(II)- and Sb(III)-oxidizing bacterium. Such processes explain why the anaerobic Sb(III) oxidation with Fe(II), Sb(III) and nitrate was more efficient than that with Sb(III) and nitrate only. Furthermore, the secondary mineral formed by strain GW3 was poorly crystalline, which is consistent with the Fe(III)- and As(V)-containing minerals produced by the thermoacidophilic Fe(II)-oxidizing archaeon *Acidianus brierleyi* (Okibe et al., 2013). The chemical composition and the adsorption efficiency of Fe(III)- and Sb(V)-containing minerals produced by strain GW3 may be further studied by changing the initial concentration of Fe(II), Sb(III) and nitrate.

Analysis of the facultative anaerobic, nitrate-dependent, Sb(III)-oxidizing strain provides an important environmental perspective for understanding the global Sb biogeochemical cycle under both oxic and anoxic environments. It may be a useful way to decrease Sb toxicity in anoxic soils and sediments. Furthermore, nitrate is considered a common contaminant in water. Thus, Sb(III) and Fe(II) oxidation coupled with denitrification by strain GW3 may also provide a potential approach for bioremediation of Sb- and nitrate-contaminated environments. In addition, strain GW3 could also oxidize As(III) under anoxic conditions, and the anaerobic catabolism of strain GW3 is most likely very variable since its genome also contained genes involved in anaerobic selenite reduction, sulfate reduction, iron reduction, and chromate reduction and transportation. Thus, this strain may provide multiple applications in bioremediation of anoxic contaminated environments.

## Author Contributions

JL designed and performed the experiments and drafted the manuscript. YZ and SZ performed the experiments. FL revised the manuscript. GW designed the study and revised the manuscript. All authors read and approved the final manuscript.

## Conflict of Interest Statement

The authors declare that the research was conducted in the absence of any commercial or financial relationships that could be construed as a potential conflict of interest.

## Footnotes

1 http://www.ncbi.nlm.nih.gov
