# Supplementary material for: Anaerobic Bacterial Immobilization and Removal of Toxic Sb(III) Coupled With Fe(II)/Sb(III) Oxidation and Denitrification
Source: Front Microbiol. 2019 Feb 27;10:360. doi: 10.3389/fmicb.2019.00360 (PMC6400856; doi:10.3389/fmicb.2019.00360)

*Frontiers in Microbiology*

**Anaerobic bacterial immobilization and removal of toxic Sb(III) coupled with Fe(II)/Sb(III) oxidation and denitrification**

Jingxin Li^1^, Yuxiao Zhang^1^, Shiling Zheng^2^, Fanghua Liu^2^, Gejiao Wang^1^*

*^1^State Key Laboratory of Agricultural Microbiology, College of Life Science and Technology, Huazhong Agricultural University, Wuhan 430070, P. R. China*

*^2^Key Laboratory of Coastal Biology and Biological Resources Utilization, Yantai Institute of Coastal Zone Research, Chinese Academy of Sciences, Yantai, Shandong, P. R. China.*


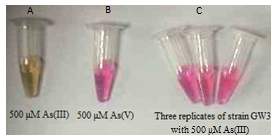


**Figure S1.** Detection of anaerobic As(III) oxidation of strain GW3. The CDM with 500 μM As(III) (A) or 500 μM As(V) (B) was used as negative or positive control, respectively. The KMnO_4_ was discolored by As(III), indicating the loss of As(III) oxidation ability. Strain GW3 cells were inoculated into CDM with 500 μM As(III) under anoxic conditions. After 4 d of cultivation, As(III) oxidation was tested with KMnO_4_ (C).


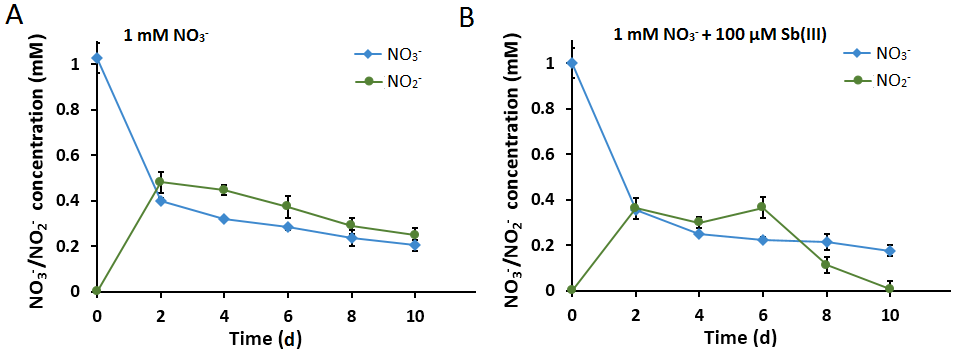


**Figure S2.** Nitrate reduction experiments of strain GW3 in CDM without Sb(III) (A) or with 100 μM (B) under anoxic conditions. For comparison, the data of the NO_3_^-^/NO_2_^-^ concentrations with Sb(III) are taken from the same experiments shown in Fig. 2A and Fig. 5C. The concentrations of NO_3_^-^/NO_2_^-^ were measured using HPLC. Error bars correspond to the standard deviations of the means of triplicates.


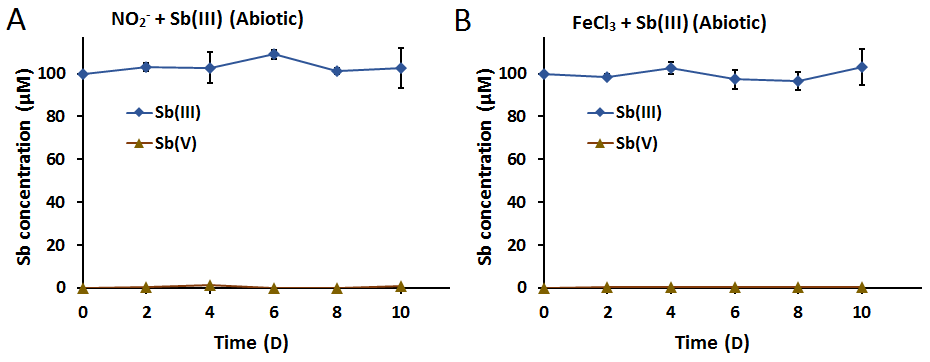


**Figure S3.** Abiotic Sb(III) oxidation with nitrite (A) and FeCl_3_ (B). One hundred micromolar Sb(III) was added to CDM containing 1 mM nitrite or 1 mM FeCl_3_, and the mixture was incubated under anoxic conditions. Error bars correspond to the standard deviations of the means of triplicates.


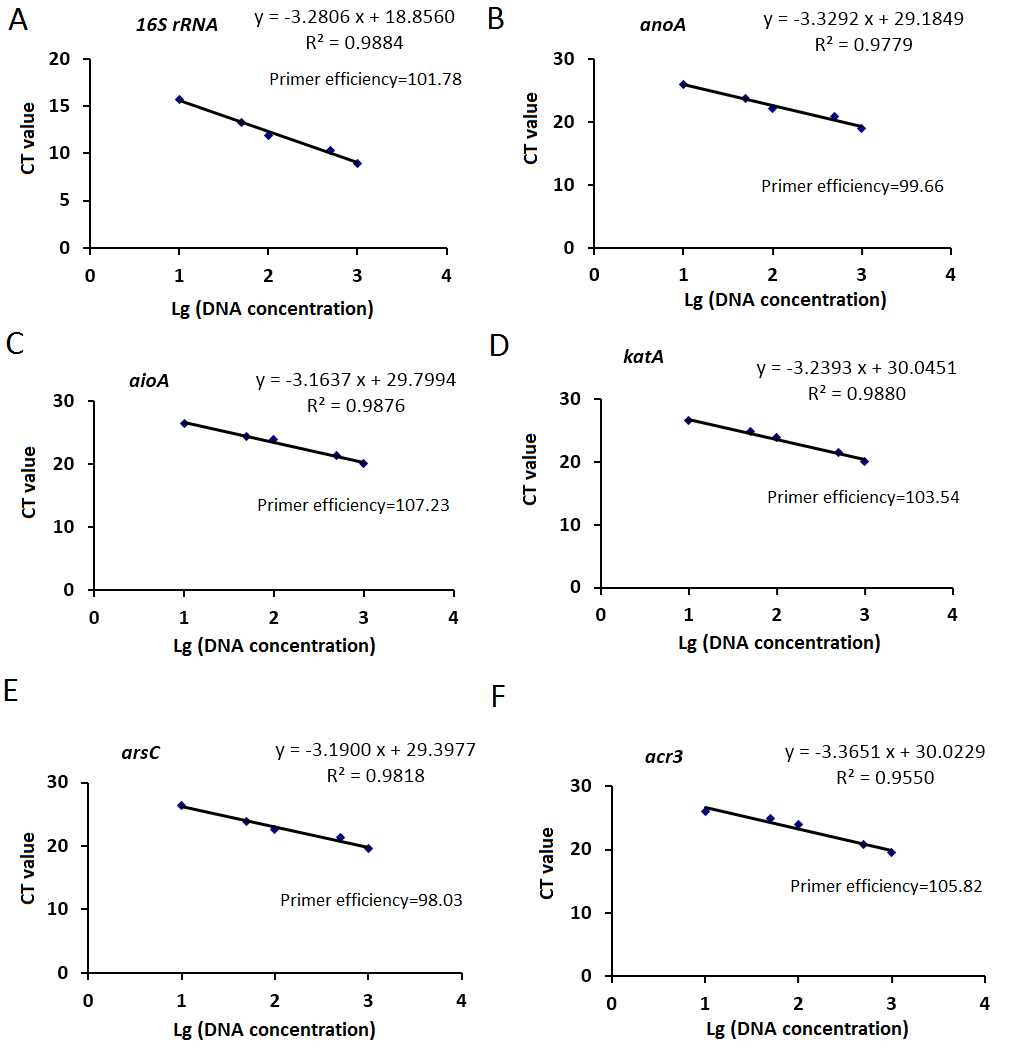


**Figure S4.** The amplification efficiency of the internal control (16S rRNA) (A) and target genes (B-F) in qRT-PCR. Genomic DNA of strain GW3 was used as a template for a series of dilutions with final concentrations of 1000, 500, 100, 50 and 10 ng/μL. Then, qPCR was conducted to obtain the CT value of each sample. The slope (S) of the standard curve was used to calculate the primer efficiency [log (1+E)=1/S].


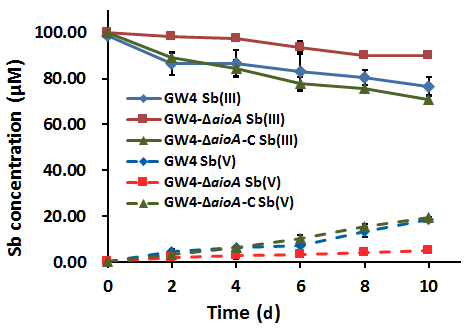


**Figure S5.** Sb(III) oxidation profiles of *Agrobacterium tumefaciens* GW4 in CDM with 100 μM Sb(III) and 1 mM nitrate under anoxic conditions. The Sb(III)/Sb(V) concentration was measured using HPLC-HG-AFS. Error bars represent standard deviations of the means from three independent experiments. Phylogenetic analysis of the 16S rRNA genes showed that strain GW4 is closely related to strain GW3 (Fan et al., 2008). In addition, the amino acid sequence similarity of AioA in these two strains are 77%.


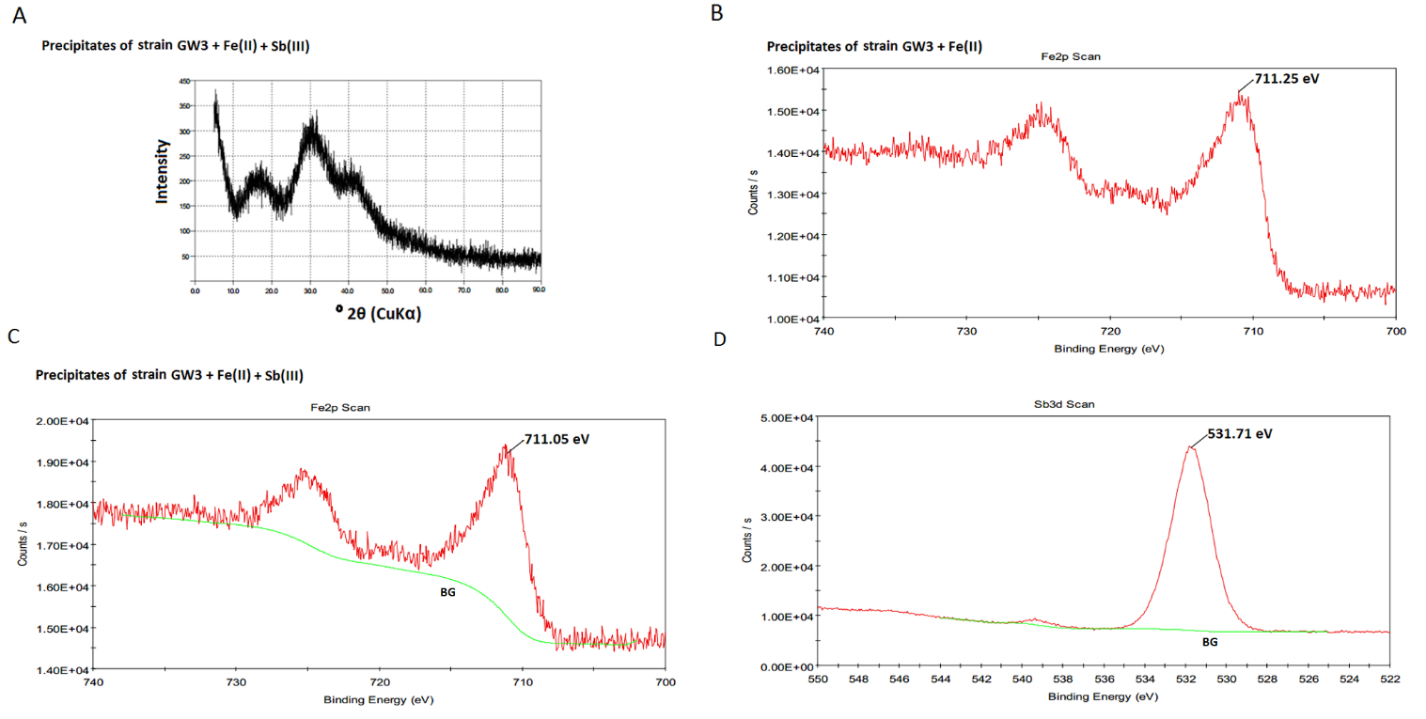


**Figure S6.** Chemical characteristics of the precipitates produced by strain GW3 in CDM medium containing 1 mM Fe(II), 1 mM nitrate and 100 μM Sb(III). (A) X-ray diffraction patterns of the secondary minerals. (B) XPS spectra of the precipitates produced by strain GW3 in CDM medium containing 1 mM Fe(II) and 1 mM nitrate. (C) XPS spectra of the precipitates produced by strain GW3 in CDM medium containing 1mM Fe(II), 1 mM nitrate and 100 μM Sb(III). The precipitates were harvested by the end of 10 days culture. The green line (BG) in C and D means the background of the XPS analysis.

**Table S1. Primers used in the present study**


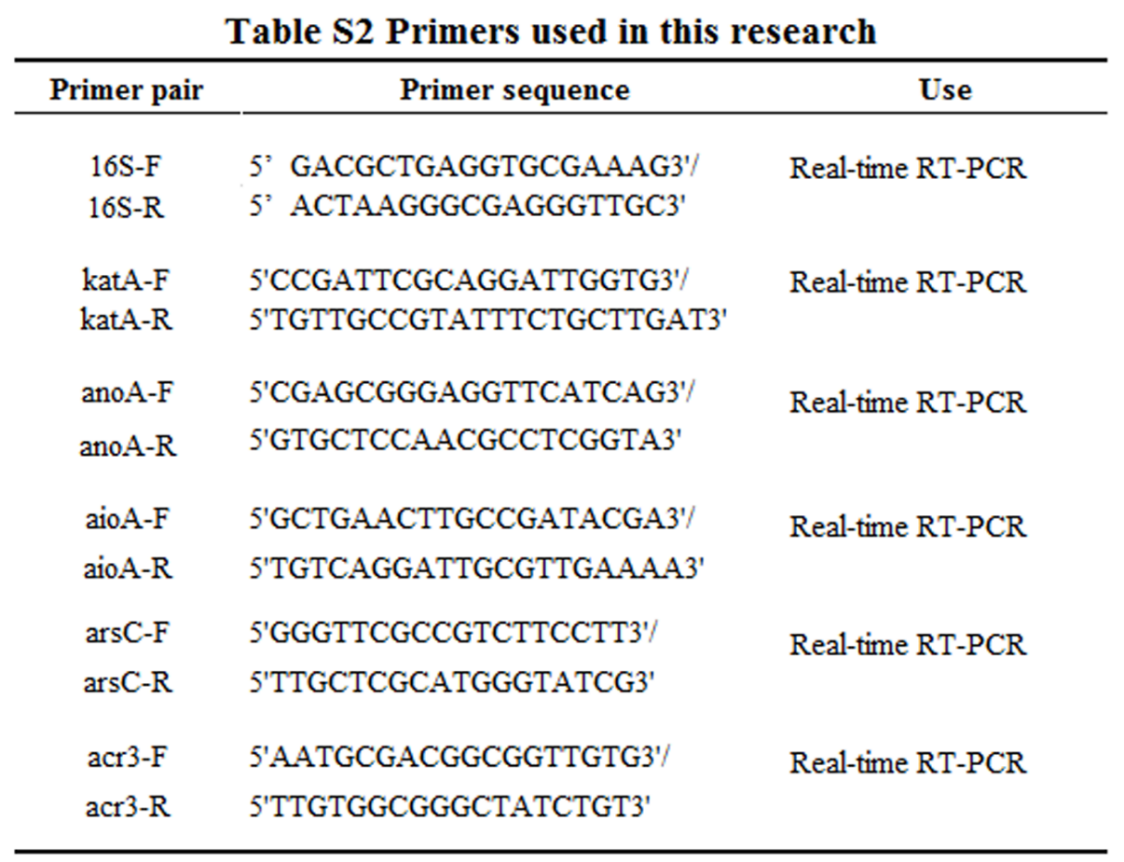


**Table S2 Putative genes involved in denitrification, Fe(II) oxidation, As(III) / Sb(III) oxidation and resistance and other anaerobic mechanisms in Strain GW3**

**
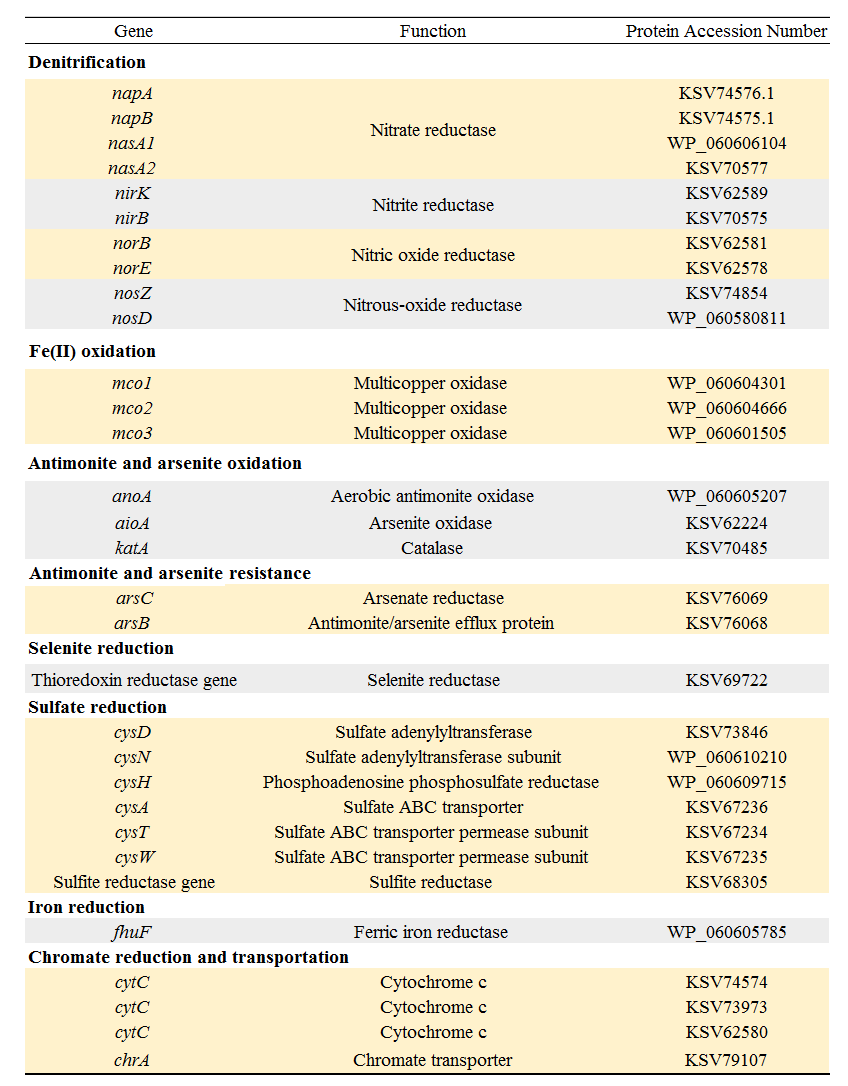
**

**Table S3 The original data of qRT-PCR under both oxic and anoxic conditions (one of the three replicates)**


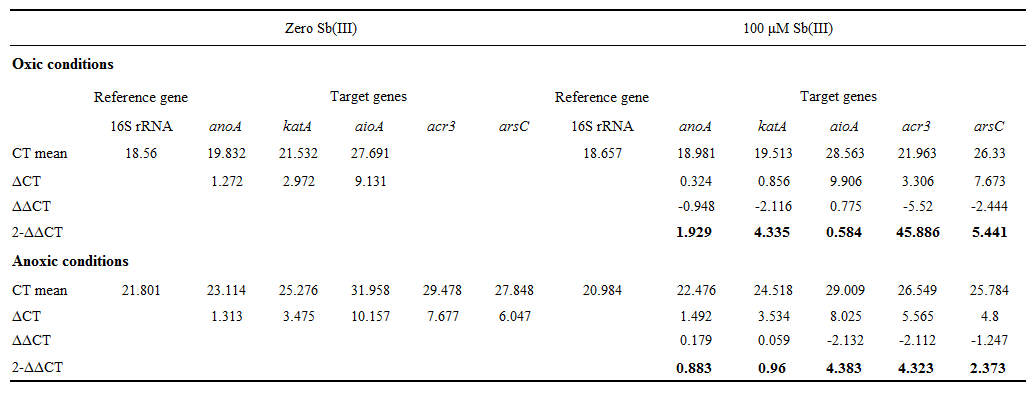

Supplement: Supplementary file 1 [file Data_Sheet_1.docx]
